# New Drugs, Appliances, and Things Medical

**Published:** 1891-01-10

**Authors:** 


### NEW DRUGS, APPLIANCES, AND THINGS MEDICAL.

[All preparations, appliances, novelties, etc., of which a notice is desired, should be sent for The Editor, to care of The Manager, 140, Strand, London, W.C.]

### ANTISEPTIC REQUISITES.

Messrs. Calvert and Co., of Bradford, Manchester, have furnished us with samples of their various antiseptic requisites. These include disinfectants in liquid and in powder; soaps for the toilet, for the washing of clothes, and for the scrubbing of ward and other floors; of a liquid dentifrice, and various other preparations. The goods sent consist of combinations of carbolic acid and phenol, with other substances in various degrees of strength. It seems almost superfluous to say anything in commendation of disinfectants and other antiseptic specialties which have been so long on the market and are so universally approved as are those manufactured by Messrs. Calvert and Co. At their request we have submitted them to the most practical tests, and have found them excellent. We may notice more particularly the drain and closet disinfectants, both liquid and in powder, and the well manufactured and agreeable toilet soaps, but most of all, the particularly pleasant antiseptic and fluid dentifrice. It is hardly necessary to say that all persons should brush their teeth. It may, however, be desirable to explain that the daily use of an antiseptic dentifrice is very desirable. We have never used a dentifrice which was more agreeable and antiseptically useful than the scented phenol preparation manufactured by Messrs. Calvert. As we said at the outset, we consider it almost a work of supererogation to commend the manufactures of such a well-known firm.

### VICHY LIQUEUR.

This is a very pleasant liqueur of the "sweet" variety, specially adapted for those whom the Vichy treatment suits. As it contains both sugar and alcohol, the method of dilution with the Vichy water will, we think, produce a pleasant variety for those gouty dyspeptics, for whom the preparation is primarily intended. It is, nevertheless, a most excellent "chasse," and one need not be gouty in order to enjoy it. Messrs. Ingram and Royle, 52, Farringdon Street, are the sole agents for its sale.

